# From Liver Fat to Cancer: Perils of the Western Diet

**DOI:** 10.3390/cancers13051095

**Published:** 2021-03-04

**Authors:** Ju Youn Kim, Feng He, Michael Karin

**Affiliations:** 1 Laboratory of Gene Regulation and Signal Transduction, Departments of Pharmacology and Pathology, University of California San Diego, 9500 Gilman Drive, San Diego, CA 92093, USA; juk005@ucsd.edu; 2 Academy of Integrative Medicine, Shanghai University of Traditional Chinese Medicine, 1200 Cailun Road, Shanghai 201203, China; fhe@shutcm.edu.cn

**Keywords:** obesity, cholesterol, NAFLD, NASH, HCC

## Abstract

**Simple Summary:**

Non-alcoholic steatohepatitis (NASH) is a common liver disease, characterized by fatty liver, chronic tissue damage, inflammation and fibrosis. NASH greatly increases the risk of the most common liver cancer, hepatocellular carcinoma (HCC), a leading cause of cancer related deaths worldwide. Here, we discuss how the Western Diet contributes to NASH and HCC development with a special emphasis on the roles of cholesterol and different metabolic regulators.

**Abstract:**

Hepatocellular carcinoma (HCC), the most common type of primary liver cancer provides the prototypical example of an obesity-related cancer. The obesity epidemic gave rise to an enormous increase in the incidence of non-alcoholic fatty liver disease (NAFLD), a condition that affects one third of American adults. In about 20% of these individuals, simple liver steatosis (hepatosteatosis) progresses to non-alcoholic steatohepatitis (NASH) characterized by chronic liver injury, inflammation, and fibrosis. In addition to liver failure, NASH greatly increases the risk of HCC. Here we discuss the metabolic processes that control the progression from NAFLD to NASH and from NASH to HCC, with a special emphasis on the role of free-non-esterified cholesterol in the process.

## 1. Introduction

Fatty liver diseases (FLD) are common liver pathologies, primarily associated with accumulation of lipids within hepatocytes, a condition known as hepatosteatosis. When liver steatosis occurs in the absence of alcohol abuse it is referred to as non-alcoholic FLD (NAFLD). FLD encompass a spectrum of pathological states, ranging from simple steatosis, characterized by profound lipid accumulation in more than 5% of hepatocytes in a given area with little or no inflammation, to non-alcoholic steatohepatitis (NASH). NASH is characterized by inflammatory infiltration along with steatosis and fibrosis, accompanied by ballooning degeneration of hepatocytes and cell death. In more advanced stages, NASH-associated fibrosis progresses to cirrhosis. Epidemiological studies indicate that approximately 25% of cirrhotic livers eventually progress to hepatocellular carcinoma (HCC), currently the 5th deadliest cancer in the U.S. In addition to increased HCC risk, NASH has become the leading cause of U.S. liver transplants. To date, more than 30% of the U.S. population is affected by NAFLD, with 20–40% of these patients progressing to NASH within six years, indicating that NASH is constantly increasing and is expected to become the leading cause of liver cancer in the near future, surpassing hepatitis virus induced HCC. NASH is strongly associated with obesity and adipose inflammation (meta-inflammation), insulin resistance, hyperinsulinemia, atherogenic dyslipidemia, and arterial hypertension [1]. Multiple parallel hits, including oxidative stress, endoplasmic reticulum (ER) stress, mitochondrial dysfunction, insulin resistance, inflammation, and gut dysbiosis has been demonstrated to be the drivers of NASH and HCC development [1,2,3,4]. Different etiologies can contribute to several of the multiple parallel hits. Liver mitochondrial dysfunction and oxidative stress caused by autophagy deficiency leads to HCC development regulated by p62 accumulation and subsequent NRF2 activation [5,6]. Mice with liver ER stress fed with high fat diet (HFD) causes liver steatosis, necro-inflammation, and ultimately develop NASH and HCC, mimicking the human features of NASH and HCC [7]. Excessive intake of fructose causes intestinal-barrier deterioration, gut microbiota imbalance, and endotoxemia that activates liver macrophage and induces liver fat accumulation, resulting in NASH and HCC [7]. Thus, HCC represents the most notable example of a cancer that is directly related to obesity and type 2 diabetes (T2D), two conditions that increase NAFLD/NASH risk. A growing body of evidence suggests that impaired cholesterol metabolism plays a crucial role in the progression from simple steatosis to NASH and from NASH to HCC, the topic on which this review is focused.

## 2. Diet and the Metabolic Syndrome

In the past 50 years the Metabolic Syndrome (MS), whose incidence closely parallels NAFLD, has reached pandemic proportions worldwide [8]. Epidemiological studies indicate that the rapid increase in the number of patients suffering from MS, an umbrella term that includes obesity, T2D, cardiovascular disease (CVD) and NASH, directly corresponds to changes in lifestyle, especially dietary patterns and sedentary behavior. In developed and rapidly developing countries, easy meals, such as ready-to-cook foods and sugar-containing soft drinks, are widely available. Although these foods make life much easier than in the past, they contain large amounts of processed and fatty meats, saturated and/or trans fats, sodium, and refined, processed sugars. Such meals provide high caloric value within a small portion, but contain small amounts or none of vegetables, fish, vitamins, and unsaturated fats. This type of diet is generally referred to as “Western diet (WD)”, implying its origin from developed Western countries. According to dietary guidelines released by the U.S. Department of Agriculture (USDA), much of the U.S. population consumes more than the recommended daily caloric value, with over 50% of the US population consuming diets that contain high amounts of saturated fat, refined sugar and sodium and far smaller portions of unsaturated fat, fiber, and vitamins than the daily recommendation. Epidemiologists found that dietary patterns are closely associated with MS incidence. Lutsey [9] and others [10] conducted a follow-up study, monitoring eating patterns and metabolic parameters of more than 3500 US and Mexican adults for several years. MS was defined according to American Heart Association guidelines as abdominal obesity, elevated fasting glucose, hypertension, and low high-density lipoprotein (HDL). When comparing the incidence of MS among participants adherent to WD with a group of prudent diet (PD) consumers, WD intake clearly increased MS risk, compared to PD consumption. In addition, this study showed that while WD intake increased the risk of MS and abdominal obesity, consumption of dairy products is preventive [10]. Ambrosini et al., tested the influence of WD on MS risk among adolescents, following dietary patterns and MS incidence in 1130 adolescents [11]. Participants were classified into the WD group, who consumed large portions of refined grains, soft drinks, red meats, and fried foods, but smaller portions of vegetables, fresh fruits, fish and nuts, or the healthy group with an eating pattern opposite to the WD. This study found that the risk of MS was increased in adolescents who consumed WD, compared to the healthy food group, especially in those with higher waist circumstance (WC) [11], suggesting that the accumulation of abdominal fats plays a pivotal role in metabolic disorders. Moreover, consuming a WD alters cholesterol metabolism and promotes low grade inflammation, manifested by elevation in circulating proinflammatory cytokines [12,13]. In contrast to WD, Mediterranean diet (MD) is rich in monounsaturated fat, fiber, and non-refined sugar and low amounts of saturated and trans fats, processed meats, and refined sugars, and is known to protect from CVD. Greeks, who mainly consume MD, appeared to be most protected from MS, among residents of seven countries, including Yugoslavia, Finland, Italy, The Netherlands, Greece, U.S., and Japan [14]. A subsequent study identified that unique ingredients, such as polyunsaturated fat (PUFA) and whole grains, protect from MS development. A diet with high PUFA to saturated fat ratio was found to increase resting metabolic rate, mitochondrial fat oxidation, and diet-induced thermogenesis [15,16], while reducing abdominal obesity, plasma cholesterol, and hepatic lipid biosynthesis [17,18,19]. Moreover, n-3 PUFA activate peroxisome proliferator activating receptors (PPAR) that inhibit the major inflammation-inducing transcription factor, NF-kB, hence carrying out anti-inflammatory and antioxidant roles [14,15,20]. Of note, NF-kB activation and two of the cytokines it induces, TNF and IL-6, play critical roles in obesity- and fat-driven HCC [2,21,22,23,24].

## 3. Pathogenesis of NAFLD and NASH

The liver is the major organ that regulates triglyceride (TG) and cholesterol (Chol) metabolism. Impaired hepatic lipid metabolism is closely linked to various aspects of the MS, including insulin resistance (IR), hyperglycemia, and hyperlipidemia, the most common metabolic dysfunctions in NAFLD patients [25]. The most prominent feature of disrupted liver lipid metabolism is steatosis, the accumulation of triglycerides. It is currently believed that fat accumulation within hepatocytes is the first hit leading to NAFLD and a prerequisite for NASH [26,27]. Hepatic lipid accumulation is determined by the balance between lipid intake, triglyceride secretion, de novo lipogenesis (DNL), and mitochondrial fatty acid (FA) oxidation. Sterol regulatory element binding protein 1c (SREBP1c) is the master regulator of FA and TG synthesis, a process involving sequential chemical reactions catalyzed by enzymes whose expression is SREBP1c-inducible. Insulin is the primary SREBP1c regulator [28]. In response to postprandial pancreatic secretion, insulin binds to the hepatocyte insulin receptor to initiate a signaling cascade that induces SREBP1c [29,30]. More than 80% of obese individuals have a fatty liver and sustained elevation of plasma insulin [31]. Furthermore, expression and activation of SREBP1c is upregulated in the livers of NASH patients [32,33], suggesting that impaired hepatic insulin signaling, and TG accumulation play pivotal roles in disease initiation and progression. Wada and coworkers used wild type (WT) and SREBP1c transgenic mice fed a high fat and high fructose diet (HFFD) and found that both mouse strains accumulated liver TG to the same extent, whereas SREBP1c transgenic mice showed a significantly increased plasma ALT, indicating that liver injury is taking place independently of TG accumulation [34]. Fatty acid synthase (FAS) and acetyl-CoA carboxylases (ACC1/2) are direct downstream targets of SREBP1c and are required for TG biosynthesis. While whole body ACC1 knockout (KO) mice are embryonically lethal, suggesting that ACC1-dependent FA and TG synthesis is indispensable to embryonic development, liver specific ACC1 KO mice are alive. As expected, ACC1 ablation significantly reduced hepatic malonyl-CoA, hepatic DNL and lipid accumulation upon sucrose feeding [35,36]. Curiously, however, Harada’s study found that the reduction in liver steatosis and DNL in the ACC1-deleted liver leads to increased plasma TG and liver injury, represented by elevated ALT and AST release [35]. The efficacy of pharmacological ACC1 inhibition has been tested in rodent models affiliated with various high caloric diets, including high sucrose [37], high fat [38], and western diet [39], and in patients with fatty liver diseases [40,41]. Administration of an ACC inhibitor remarkably reduced hepatic steatosis and DNL, consistent with the effect of ACC1 ablation. Intracellular ACC regulator that mimics ACC inhibitor is monophosphate-activated protein kinase (AMPK), an energy sensor, that is activated upon glucose deprivation, obesity, or starvation [42]. AMPK phosphorylates ACC at Ser^79^, thereby suppressing production of malonyl-CoA. Thus, AMPK activation directs acetyl-CoA to the TCA cycle to produce ATP [42]. Indeed, a mouse which harbors point mutation at Ser^79^ (ACC KI) showed increased hepatic lipogenesis, compared to WT mice, upon fructose intake [43]. Moreover, ACC KI mice show increased tumorigenesis in response to carcinogen-induced liver injury, underlining the potentials of AMPK inhibitors, metformin, as preventative therapeutics for NASH and HCC [44]. However, genetic ablation or pharmacologic inhibition of ACC resulted in increased serum TG, reduced hepatic polyunsaturated fatty acids (PUFA) and increased SREBP1 and its downstream lipogenic enzymes [37,39,40]. Moreover, long-term ACC inhibition perturbs hepatic glucose homeostasis [37], leading to hyperglycemia that aggravates the condition of patients with fatty liver diseases. While ACC is suppressed, acetyl CoA participates in regulation of gene transcription and protein modification by histone and protein acetylation [45], respectively. For instance, FoxO1, a key regulator of gluconeogenesis, is stabilized in the nucleus by acetylation [46]. Therefore, the utility of ACC inhibition in NASH and HCC needs a more careful and long-term evaluation. Global FAS ablation also caused embryonic lethality [47], but liver specific FAS KO mice showed elevated hepatic steatosis following high carbohydrate intake [48]. Carbohydrate response element binding protein (ChREBP) is the transcription factor that stimulates lipogenesis in response to glucose intake by directly inducing the same lipogenic enzymes that are induced by SREBP1c [49]. ChREBP ablation reduces liver and serum TG in response to high starch intake but increases accumulation of free cholesterol (FC), ER stress, cell death and fibrosis [50,51]. TG synthesis depends on the esterification of diacyl glycerol (DAG) by FA, a reaction catalyzed by diacylglycerol acyl transferase (DGAT). Yamaguchi and colleagues suppressed DGAT2 by injecting antisense oligonucleotides (ASO) into obese db/db mice and found that impaired TG synthesis increased hepatocyte death and inflammation, indicating that TG accumulation protects hepatocytes from liver injury and progression to NASH [52]. It should also be noted that despite being obese and hepatosteatotic, db/db mice never develop NASH. These results strongly support the hypothesis that the progression from NAFLD to NASH depends on multiple secondary hits that result in hepatocyte death and liver inflammation [27,53]. Most commonly proposed secondary hits include mitochondrial dysfunction and ER stress, but the triggers that initiate them remain unknown.

## 4. Cholesterol and NASH Pathogenesis

Hepatic Chol metabolism includes Chol biosynthesis, secretion, and excretion. Hepatocytes synthesize Chol through the coordinated action of multiple enzymes, whose expressions are regulated by SREBP2 [47]. Elegant and fundamental work from the Goldstein and Brown group showed that SREBP2 activation is tightly controlled by the amounts of sterols in the ER lumen. Once luminal Chol declines, SREBP cleavage activating protein (SCAP) is released from sequestration by INSIG at the ER membrane and escorts SREBP2 to the Golgi apparatus, where it is sequentially cleaved by site-1 protease (S1P) and site-2 protease (S2P), resulting in of release its cytoplasmic facing N-terminal activation domain that enters the nucleus to induce Chol biosynthetic enzymes [48]. A similar process leads to SREBP1 cleavage-mediated activation. Interestingly, while the plasma membrane is the site where most intracellular Chol resides, the ER is the site at which Chol shortage is sensed. Accordingly, ER stress and the unfolded protein response (UPR) also control Chol biosynthesis by regulating SREBP2 cleavage [54]. However, the underlying mechanism remains controversial [55,56]. In addition to its role in Chol biogenesis, SREBP2 induces expression of enzymes required for formation of very low-density lipoproteins (VLDL) [49]. VLDL formation is initiated by apolipoprotein B (Apo B) lipidation at the rough ER and by moving from the ER to the Golgi apparatus, Apo B matures by combining with core VLDL, composed of TG and cholesterol-esters (CE), resulting in generation of VLDL. Wang and colleagues had shown that the ER stress and UPR effector IRE1 controls activation of ER-resident lipidation enzymes, supporting Apo B maturation and formation of VLDL [50]. However, we [3] and others [57] found that IRE1 stimulates the translation of caspase-2, which through S1P cleavage leads to SREBP1/2 activation and lipid and Chol biosynthesis. Increasing the amount of hepatic lipids alters the ratio of FC to CE at the core VLDL but the number of VLDL particles remains unchanged, whereas elevated SREBP2 activity increases VLDL formation. Accordingly, inhibition of the SREBP2 target HMG-CoA reductase (HMGCR) by statins suppresses VLDL formation and secretion, indicating that these two pathways are interconnected [58]. Secretion of conjugated sterols from hepatocytes as bile acids is another pathway that regulates liver Chol homeostasis. Bile acids are produced by the initial modification of Chol by CYP7A1 and further modification by oxidative enzymes located within microsomes, peroxisomes and mitochondria [59]. Once bile acids are formed, they are excreted from the hepatocyte through canaliculi receptors, ABCA11 and ABCG5/8, to the intestine where they facilitate lipid absorption and also undergo secondary modifications by bacterial enzymes. Bile acids or their precursors regulate hepatic Chol homeostasis by acting as ligands for nuclear receptors. One such receptor, liver X receptor (LXR), a major activator of SREBP1c, binds oxysterol and in turn induces expression of ABCG5/8 to increase biliary Chol secretion [60]. Farnesoid X Receptor (FXR) is activated by bile acids and coordinates adaptive responses to altered bile acid metabolism. FXR activation by the bile acid chenodeoxycholic acid (CDCA) or synthetic ligands ameliorates inflammatory responses through repression of NF-kB activity and subsequent inhibition of cytokine expression [61,62]. Altered Chol metabolism is implicated in NASH pathogenesis. FC deposition is modestly increased in a fraction of NAFLD patients with simple steatosis but is markedly elevated in all NASH patients [62,63]. TG accumulation, detected by oil red O (ORO) staining, is higher in NAFLD relative to NASH, but elevated hepatic FC correlates much better with ballooning degeneration and liver fibrosis [63,64]. Lipidomic analysis of liver biopsies from NAFLD and NASH patients also found that FC accumulation positively correlates with NASH, whereas simple steatosis is inversely correlated with FC, despite showing increased TG [52,65]. FC deposition is found to cause cellular toxicity, including mitochondrial dysfunction and ER stress, the two main secondary hits that lead to NASH, thus increasing NASH risk and severity [66]. In addition to enhanced conversion of CE to FC, upregulation of Chol biosynthesis, and downregulated Chol excretion have also been observed in the NASH liver [67,68]. 

**Figure 1:**
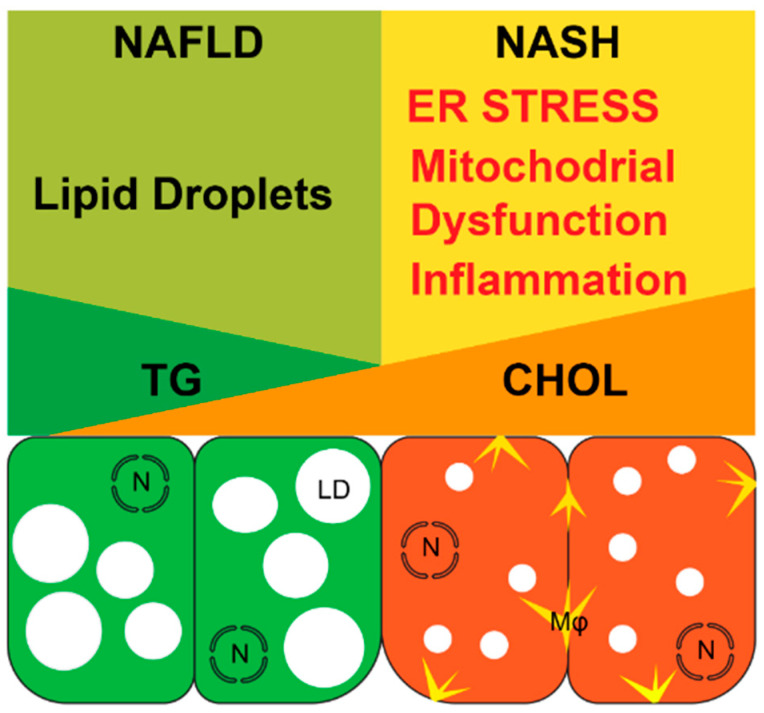
The key features that distinguish NAFLD from NASH. N: Nucleus, LD: Lipid Droplet, Mφ: Macrophages.

Statins are widely used drugs that lower plasma Chol and thereby reduce CVD risk [69]. Randomized controlled trials (RCT) indicate that statin use may have a favorable effect on the risk of NAFLD aggravation. However, the effect of statins on NASH and HCC is inconclusive, because many patients who are on statins have many other confounding factors, including MS, heavy alcohol intake, hepatitis virus infections, and other medicines. Nonetheless, studies were conducted with elderly or diabetic cohorts, who use or do not use statins, to assess the effect of statins on NASH progression. After exclusion of individuals with confounders, it was found that statin use was inversely correlated with NASH and fibrosis scores, with protective effects on patients who have a body mass index (BMI) higher than 27.5 [61,62]. In another population based study, researchers analyzed the incidence of hepatic steatosis among 2578 subjects who underwent ultrasonography and found that patients who have been on statins for more than 2 years showed an inverse correlation with steatosis and HCC [70]. Another study conducted histological analysis of NASH biopsies from patients who used or did not use statins found that statin use decreased the risk of steatosis, NASH and lowered the fibrosis score [71]. These clinical studies strongly suggest that Chol biosynthesis is positively associated with NAFLD severity, NASH and progression to HCC (Figure 1).

## 5. NASH Mouse Models

### 5.1. Diet-Induced NASH Models

Methionine and choline deficient (MCD) diet has been widely used to study NASH pathogenesis. This diet is high in sucrose (40%) and has a modest amount of fat (10%) but lacks methionine and choline. After eight weeks of MCD-feeding C57BL6 mice exhibit massive liver steatosis, inflammatory infiltration, and fibrosis, which are some of the classical signs of human NASH. One advantage of the MCD diet model is the rapid onset of hepatic steatohepatitis. However, the major drawback is that the metabolic parameters induced by MCD diet intake are opposite to those of human NASH, including decreased body weight, lack of obesity, low serum TG and Chol levels, and the absence of IR [72]. Importantly, methionine and choline are essential components for generation of phosphatidylcholine (PC), which is required for mitochondrial membrane function and VLDL assembly [73]. Due to the absence of PC, the MCD-fed liver presents with elevated lipid deposits and oxidative stress. To avoid substantial weight loss, a choline-deficient, L-amino acid defined (CDAA) diet, which contains methionine, has been used. In rats and mice, the CDAA diet induces liver steatosis, inflammatory infiltration, and fibrosis, similar to the MCD diet, but their onset is slower compared to the one caused by MCD feeding [68,74,75]. The CDAA diet results in induction of hepatocellular adenoma and carcinoma within 84 weeks of feeding, with 16% and 5% incidence in male mice, respectively [74]. Despite methionine supply, the choline deficiency suppresses TG secretion and dramatically inhibits hepatic Chol biosynthesis, which is increased in patients with steatohepatitis [76]. 

High fat diet (HFD) is the most commonly used diet to induce FLD in animal models. HFD derives 60% of its caloric value from saturated fat and HFD feeding of C57BL6 mice for several months results in simple steatosis, adipose tissue expansion, IR, and hyperglycemia. However, HFD barely induces hepatic inflammation, ballooning hepatocyte degeneration, and fibrosis [72]. Various diet combinations have also been used. For instance, the HFD-CD diet is composed of 60% saturated fat, supplemented by L-amino acids, but no choline. HFD-CD consumption results in liver steatosis with substantial inflammation and some fibrosis within 6 months and unlike HFD, it induces liver tumorigenesis within 12 months, indicating that the absence of choline initiates tumorigenesis in the presence of HFD-induced steatosis. Interestingly, HFD-CD diet leads to activation of SREBP2, suggesting that the choline deficiency stimulates a signaling pathway linked to non-canonical hepatic SREBP2 activation [68,77]. Fructose is a highly lipogenic carbohydrate. Accordingly, high fructose diet (HFrD) is often used to study the role of hepatic DNL in FLD pathogenesis [78]. HFrD intake causes liver steatosis by increasing DNL, resulting in elevated serum TG and Chol. Unlike high sucrose diet, prolonged HFrD intake results in appearance of MS, glucose intolerance and IR [7,79]. Despite these metabolic dysfunctions, which depend on barrier disruption and chronic endotoxemia, HFrD feeding does not result in liver injury, inflammation or fibrosis, and no progression to HCC, unless this diet is given to inflammation-prone MUP-uPA transgenic mice or combined with a chemical carcinogen [7]. A combined high fat high carbohydrate diet (HFHC), containing fructose was found to induce steatosis, IR, liver injury, extensive inflammation and fibrosis in C57/BL6 mice, within 16 weeks of feeding [80].

### 5.2. Genetic Models

#### 5.2.1. SREBP Transgenic Mice

Three SREBP isoforms have been identified, of which SREBP1a and 1c are transcribed from a single gene and preferentially stimulate DNL [81], whereas SREBP2 is encoded by a distinct gene and mainly regulates expression of genes involved in Chol synthesis and transport [82]. Liver-specific SREBP-1c transgenic mice develop simple steatosis, increased visceral adipose tissue, hypertriglyceridemia, and hyperinsulinemia, but no significant inflammation by 24 weeks of age [83]. Adipose-specific SREBP-1c transgenic mice display spontaneous hepatic steatosis, hyperglycemia, hypertriglyceridemia, insulin resistance and diabetes at the age of 8 weeks [84] and at 20 weeks of age, their livers show typical NASH features, pericellular fibrosis, ballooning degeneration, and Mallory Denk bodies [84]. However, these mice show reduced peripheral fat due to defects in adipocyte differentiation and exhibit low levels of circulating leptin [84]. Liver-specific SREBP2 transgenic mice show Chol and FA synthesis, but no increase in plasma cholesterol [85]. Intestinal-specific SREBP2 expression also increases Chol and FA synthesis, but this time Chol and TG in liver and intestine are increased [86].

#### 5.2.2. db/db and ob/ob Mice

The db/db mouse has a point mutation in the leptin receptor gene, whereas ob/ob mice harbor a mutation in the leptin gene, resulting in a defective leptin signaling [87]. Both mice are hyperphagic, obese, and diabetic, showing severe hyperglycemia, hyperinsulinemia, and macrovesicular hepatic steatosis, but no inflammation and fibrosis, key features of NASH [88]. However, secondary hits, including MCD diet, ethanol, endotoxin, or high-iron supplementation induce steatohepatitis in these mice [88]. However, congenital leptin receptor deficiency and leptin resistance caused by gene mutations in obese or NASH patients are rare [88,89], so db/db and ob/ob mouse models are limited in their ability to reflect the etiology of human NASH. 

#### 5.2.3. Fat Aussie (foz)/foz Mice

foz/foz mice are deficient for Alms1, which encodes a ubiquitous protein localized at the basal bodies of cilia that plays a role in intracellular trafficking and whose deficiency is responsible for Alström syndrome [90]. foz/foz mice are hyperphagic, and obese, but the severity of NASH symptoms depends on their genetic background. foz/foz C57BL6/J mice develop spontaneous steatosis, hyperinsulinemia, ballooning hepatocyte degeneration, and hepatic inflammation, while foz/foz BALB/c mice do not [91].

#### 5.2.4. KK-A^y^ Mice

KK-Ay mice have a heterozygous mutation of the agouti gene (KK-Ay/a), which encodes a paracrine signaling molecule that causes hair follicle melanocytes to synthesize the yellow pigment pheomelanin instead of the black or brown pigment eumelanin [92]. These mice exhibit a yellow fur and are hyperphagic due to impaired hypothalamic appetite suppression. As a result, these mice develop typical hepatic steatosis, insulin resistance, hyperglycemia, hypertriglyceridemia, and hyperleptinemia [93,94]. However, these mice do not develop NASH spontaneously. When these mice are fed with MCD, they exhibit a strong NASH phenotype, including steatohepatitis, and profibrogenic responses [94]. 

#### 5.2.5. Major Urinary Protein (MUP)-Urokinase-Type Plasminogen Activator (uPA) Mice

MUP-uPA mice express uPA protein in hepatocytes under control of the MUP promoter [95]. uPA accumulates in the ER and results in transient hepatocyte ER stress and liver injury in 5 to 6 week-old MUP-uPA mice, which is accompanied with hepatocyte repopulation and transgene extinction, resulting in gradually attenuated liver injury [2]. Although these mice develop hepatosteatosis at five weeks due to ER-stress mediated caspase 2 activation and subsequent SREBP1/2 activation, adult MUP-uPA mice do not show hepatic steatosis [3]. However, in response to HFD feeding MUP-uPA mice develop steatohepatitis, pericellular and bridging fibrosis, resembling the pattern in human NASH, including hepatocyte ballooning and accumulation of Mallory Denk bodies, together with body weight gain and insulin resistance [2]. After eight months of HFD feeding, 90% of these mice show large HCC nodules. 

#### 5.2.6. Liver-Specific Phosphatase and Tensin Homologue (PTEN) Deleted Mice

PTEN is a tumor suppressor gene encoding a lipid phosphatase, whose major substrate is phosphatidylinositol-3,4,5-triphosphate. Thus, PTEN is a negative regulator of the phosphatidylinositol 3-kinase/AKT signaling pathway responsible for many of the metabolic actions of insulin [96]. Liver-specific PTEN knockout mice show hepatomegaly and steatosis at 10 weeks of age, and steatohepatitis with fibrosis at 40 weeks of age [97]. Although these mice exhibit almost the same histological features as human NASH, PTEN deletion in liver causes liver insulin hypersensitivity with improved systemic glucose tolerance [96]. 

#### 5.2.7. Liver-Specific NF-κB Essential Modulator (NEMO) Deficient Mice

NEMO also known as inhibitor of NF-κB kinase subunit γ (IKKγ), is required for NF-κB activation in response to a variety of stimuli by phosphorylating IκB proteins, leading to their ubiquitination and degradation [98]. Liver-specific NEMO knockout mice exhibit spontaneous liver damage, hepatosteatosis, and fibrosis resembling human NASH at six months old of age [4]. However, these mice do not show obvious obesity or metabolic syndrome, but they do progress to HCC [96].

#### 5.2.8. Special Diet-Induced Animal Model of Non-Alcoholic Fatty Liver Disease (DIAMOND) Mice

DIAMOND mice are derived from a cross of two common mouse strains, 129S1/SvImJ and C57BL/6J [99]. When DIAMOND mice are fed with HFD accompanied by ad libitum consumption of water with a high fructose and glucose content (Western diet sugar water (WD SW)), they develop steatosis (4–8 weeks), steatohepatitis (16–24 weeks), progressive fibrosis (16 weeks onwards), together with obesity, insulin resistance, hypertriglyceridemia, and increased LDL-cholesterol [99]. These mice mimic all of the physiological, metabolic, histological, transcriptomic gene signature, and clinical endpoints of human NASH [99]. It is interesting that only the B6/129 hybrid, but not the parental strains, that recapitulates all aspects of human NASH. 

#### 5.2.9. Low Density Lipoprotein (LDL) Receptor (LDLR) Knockout Mouse 

LDLR is a cell surface-glycoprotein that mediates the uptake of excess circulating LDL Chol by hepatocytes, where the Chol is further catabolized and eventually secreted in the feces via the bile [100]. *LDLR*-deficient mice show reduced hepatic LDL clearance, elevated plasma LDL, which accelerates atherosclerosis and hence are commonly used as models for atherosclerosis [100]. HFD feeding of *LDLR*-deficient mice leads to macrosteatosis and hepatocyte ballooning, with no significant inflammatory infiltrates [101]. These mice develop early hepatic inflammation and steatosis when fed a high-fat-high-Chol (HFC) diet [100], suggesting that dietary Chol, rather than liver steatosis, leads to hepatic inflammation in hyperlipidemic mouse models of NASH [102]. However, *FXR* and *LDLR* double knockout mice show occasional foci of inflammatory cells when fed a control diet, that are greatly increased after HFD feeding resulting in extensive inflammation and ballooning degeneration of hepatocytes that resembles human NASH [101]. FXR is the primary sensor for endogenous bile acids and plays a crucial role in hepatic triglyceride, bile acid, and glucose homeostasis by regulating the expression of various metabolic genes [103]. *FXR* single knockout mice have mild steatosis and fibrosis due to the chronic liver damage, but not a pronounced NASH phenotype [104].

## 6. Cholesterol and HCC

Chol plays important roles in maintaining cell membrane fluidity and permeability, as well as modulating the tightly controlled network of intracellular signaling and lipid transfer. Chol enters the circulation either by intestinal absorption of Chol containing diets, or de novo synthesis primarily by the liver. Chol is stored in the liver as CE or assembled into VLDL, together with triglycerides and apolipoproteins, as well as high-density lipoprotein (HDL) particles in the blood. HCC is a complex disease with a variety of known etiologies, including virus infections, NASH, ASH, hemochromatosis, primary biliary cirrhosis, genetic mutations, and exposure to toxicants such as triclosan, aflatoxin B1, diethylnitrosamine (DEN), polyvinyl chloride, and carbon tetrachloride [105]. Strong evidence has emerged that FC is a major risk factor for NASH and HCC and a potential common cross-etiological factor that drives both inflammation and liver cancer. In addition, HCC patients are frequently with other liver diseases such as chronic hepatitis and cirrhosis, which impair the hepatic cellular functions including cholesterol metabolism, resulting in altered levels of plasma lipids, Chol, lipoproteins, and apolipoproteins reflecting patients’ pathologic conditions. Several studies reported that high serum Chol and high-density lipoprotein-cholesterol (HDL-C) levels are negatively associated with HCC incidence [106,107,108] and mortality [109,110]. Decreased serum Chol level was associated with decreased disease-free survival (DFS) and overall survival (OS) of HCC patients [110]. Decreased serum Chol could also be due to the increased consumption of Chol by the tumor cells, as tumor tissues contain increased amounts of cholesterol for their proliferating metabolic demands when compared with the corresponding normal tissues [111,112]. Furthermore, high serum levels of Chol lead to increased accumulation of Chol in the natural killer cells and activates their antitumor functions to reduce the liver tumor growth [113]. However, other studies suggested that increased serum cholesterol level is positively correlated with tumor aggressiveness of HCC [114,115]. It is clear that obesity and metabolic syndromes increase the HCC risk. These discrepancies might reflect the differences in liver disease stages and etiologies, where high serum Chol levels may overlap but not cover all key risk factors, such as insulin resistance, immune dysfunction, and obesity. Dietary Chol has been shown to play a role in the development of NASH and HCC in both animal models and human [88,116], and epidemiological studies indicate that Chol intake is an independent risk factor for HCC [114,117]. FC accumulation in hepatocytes leads to hepatocyte injury, macrophage recruitment, liver fibrosis, and HCC development [118]. Mice fed high-fat high Chol diet (HFHCD) show NASH development, whereas animals fed high-fat without Chol diet (HFD) develop simple steatosis [119]. In addition, compared to HFD, HFHCD increases HCC tumor burden in mice exposed to DEN [119].

### 6.1. Cholesterol Homeostasis Is Dysregulated in NASH

Insulin resistance or hyperinsulinemia accompanied by NAFLD induce alterations of hepatic Chol metabolism, resulting in hepatic accumulation of FC [116,120]. NAFLD patients show increased Chol biogenesis due to SREBP2 activation, elevated Chol overload via LDLR upregulation, while reduced biotransformation of Chol to bile acids due to reduced expression of CYP7A1, the rate limiting enzyme for bile acid synthesis, as well as decreased excretion of bile acids and Chol due to downregulation of ABCB11, ABCA1, and ABCG8 [63,120]. In addition, NAFLD patients show increased expression and activity of HMGCR, a rate-limiting enzyme in the mevalonate pathway that catalyzes the first reaction in Chol biosynthesis [120]. Many foods consumed by humans contain high levels of Chol and dietary Chol exacerbates hepatic accumulation of free cholesterol [116]. Recent studies showed that intracellular Chol transport is also altered in NAFLD and NASH, including increased expression of a main caveolae scaffold protein caveolin-1 involved in Chol-binding and transport, steroidogenic acute regulator (StAR)-related lipid transfer proteins involved in Chol transport from intracellular stores to the mitochondria, and oxysterol-binding protein (OSBP), decreased expression of Niemann–Pick C1 (NPC1) involved in the egress of Chol from the endosomal/lysosomal compartment [63,121].

### 6.2. Cholesterol Toxicity Drives Liver Injury, Inflammation, Fibrosis, and HCC Development

Altered Chol metabolism exerts toxic effects on hepatocytes, Kupffer cells (KCs), and hepatic stellate cells (HSCs) through diverse mechanisms. Cellular Chol overload activates KCs in mice fed with HFHCD and FC accumulation in KCs is necessary for their conversion to a proinflammatory phenotype and NASH development [122]. In addition, uptake of oxidized LDLs (oxLDLs) by KCs through the scavenger receptors cluster of differentiation 36 (CD36) and scavenger receptor A (SR-A) leads to KC activation and liver inflammation [123]. Intracellular FC overload into HSCs through the lectin-like oxidized LDL receptor-1(LOX-1) activates Toll-like receptor (TLR)-4-dependent pathway and triggers hepatic fibrosis in NASH [124,125].

FC accumulation in ER triggers ER stress by altering the critical Chol-to-phospholipid ratio of the ER membrane, which stiffens ER membrane and impairs ER protein function and protein folding capacity [126]. Increased ER Chol inhibits sarco/ER calcium ATPase (SERCA) activity and causes the depletion of ER calcium [127]. ER stress causes the generation of reactive oxygen species (ROS) and oxidative stress, which activates nuclear factor erythroid 2-related factor 2 (NRF2) [128,129]. In addition, ER stress triggers a compensatory adaptive response, the unfolded protein response (UPR), orchestrated by three ER transmembrane receptor proteins inositol requiring kinase 1 (IRE1), protein kinase R (PKR)-like endoplasmic reticulum kinase (PERK), and activating transcription factor 6 (ATF6) [130]. In addition to the IRE1-caspase 2 pathway, IRE1-XBP1s signaling induces lipid synthesis and liver-specific XBP-1 deficient mice decrease hepatic lipogenesis followed by inhibited lipid accumulation following a lipogenic diet [56]. In the event of chronic damage or when the stress is too severe for restoration of ER function, the UPR induces cell death [130]. Both NRF2 and UPR PERK-ATF4 signaling promote tumor growth by reprogramming tumor metabolism [129,131,132].

Chol accumulation in mitochondria results in altered membrane fluidity and mitochondrial dysfunction, including impairment of the 2-oxoglutarate carrier for mitochondrial transport of GSH, an essential antioxidant that maintains mitochondrial redox homeostasis [132]. In primary mouse hepatocytes mitochondrial Chol deposition activates mitochondrial membrane pore transition and ATP depletion, as well as hepatocyte apoptosis and necrosis by activating JNK1 [133,134]. JNK1-mediated cell death also results in the release of high mobility group box 1 (HMGB1) protein, which in turn activates TLR4 in neighboring hepatocytes to enhance liver inflammation and injury [135] These dysfunctions of the ER and mitochondria induced by FC accumulation contribute to liver injury and HCC development through compensatory proliferation. 

**Figure 2:**
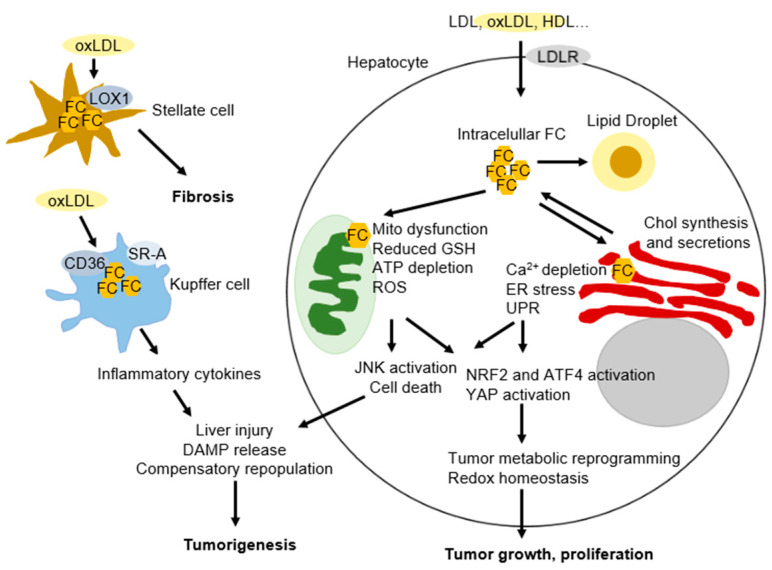
FC induces HCC formation and development. In hepatocytes, cholesterol, mainly in the forms of LDL, oxLDL, HDL, and chylomicron remnant, is internalized via LDLR-mediated endocytosis and cholesterol esters are then hydrolyzed by acid lipase enzymes in the endosomes and/or lysosomes, ultimately leading to the elevated intracellular FC pools. FC is transported to various organelles, such as mitochondria (Mito), endoplasmic reticulum (ER), and lipid droplets, via different protein carriers. Overload of FC into ER causes ER calcium depletion, ER stress, and sequential adaptive response-unfolded protein response (UPR). Accumulation of FC into Mito leads to Mito dysfunction including reduced GSH levels in the Mito, ATP depletion, and generation of ROS. If the stress in the ER and Mito induced by FC are too severe or prolonged, ER and Mito stress trigger cell death and subsequent release of damage-associated molecular pattern (DAMP) including HMGB1 and oxidized Mito-DNA, resulting in liver injury and compensatory proliferation. In addition, cholesterol uptake in the hepatic stellate cells by lectin-like oxidized LDL receptor-1(LOX-1) triggers stellate cell activation and fibrosis. Internalization of cholesterol into Kupffer cells by CD36 or scavenger receptor A (SR-A) leads to Kupffer cells activation and release of proinflammatory cytokines, which also contributes to liver injury, inflammation and HCC initiation. Furthermore, FC-mediated ER and Mito stress activates NRF2 and ATF4, two key transcription factors in the stress response to mediate the survival of stressed hepatocytes. oxLDL uptake into cancer cells promotes lipophagy and enhances YAP oncogenic activity. If the stress is reparable, activated NRF2, ATF4, and YAP maintain the cancer cell redox homeostasis and reprogram metabolism for the HCC growth and proliferation.

In NASH-associated tumors from both murine models and clinical specimens, cancer cells show uptake of oxidized low-density lipoprotein (oxLDL) via CD36, which triggers expression of CCAAT/enhancer-binding protein β (C/EBPβ) to upregulate an ER-resident protein, NogoB, that interacts with ATG5 to promote lipophagy, leading to lysophosphatidic acid-enhanced YAP oncogenic activity [136] (Figure 2).

### 6.3. Cholesterol Modulators in HCC Therapy

Dysregulation of hepatic Chol metabolism in NASH and HCC pathogenesis and progression provides attractive opportunities for pharmacological restoration of Chol homeostasis. Weight loss is the most effective approach to prevent obesity and fatty liver diseases. Adiponectin, an adipokine released from fat, was found to increase in-parallel with the severity of obesity, and considered as a key molecular regulator of fat hyperplasia and hypertrophy but to decrease in patients with NASH and HCC, implicating involvement in lipotoxicity and inflammation [137]. Cumulative studies indicate that adiponectin elicits beneficial effects that oppose lipotoxicity, including suppression of TNF and NF-κB activation along with elevation of energy expenditure [138]. Adiponectin also activates AMPK and PPARα and recently it was shown that AMPK phosphorylates HMGCR and suppresses Chol synthesis [139]. Chol synthesis inhibition with statins could have beneficial effects on NAFLD [140]. Statins, however, have not been widely adopted in NAFLD/NASH therapy, in part because of concerns about potential liver toxicity [141]. Recent studies show that statins can be safely administered to patients with NAFLD, including those with elevated transaminase levels [142] and the use of statins in patients with elevated plasma aminotransferases may be beneficial [143]. Post hoc data from three large prospective randomized clinical trials suggest that specific statins (mainly atorvastatin) ameliorate NAFLD/NASH and reduce CVD events. Several biopsy studies have found that rosuvastatin use is related with significant histological ameliorating effects in the setting of NASH. Statin treatment may also protect from HCC [143]. In contrast to the positive impact of statins on mortality in the general population, another report showed that the use of statins and other LLAs did not reduce the increased risk of overall or cardiovascular mortality in NAFLD [144]. Thus, statin use for the treatment of NASH/HCC awaits further evaluation.

Ezetimibe, a Chol lowering agent that reduces intrahepatic Chol by inhibiting Chol absorption from the intestine, modestly lowers serum Chol and clinical data suggest that ezetimibe may be beneficial for NAFLD [140]. A meta-analysis showed that ezetimibe attenuated serum liver enzymes and hepatic steatosis and ballooning in six studies, but it only improved hepatocyte ballooning and not hepatic inflammation and fibrosis in patients with NAFLD and NASH [145]. Further well-designed randomized and fully powered trials are needed to establish the role of ezetimibe in NASH and HCC.

Bile acid sequestrants (BAS) are orally administered polymers that bind bile acids in the intestine forming nonabsorbable complexes [140]. BAS interrupt intestinal reabsorption of bile acids and decrease their circulating levels. In a Western diet-induced NASH mouse model, the BAS sevelamer reversed liver injury and prevented progression of NASH [146]. This beneficial effect was associated with reversing microbiota complexity in cecum [146]. However, current clinical data do not support this contention.

FXR agonists are a group of FXR-activating ligands that modulate carbohydrate and lipid metabolism and insulin sensitivity [147]. The FXR agonist obeticholic acid showed beneficial effects in patients with steatohepatitis in a multicenter, randomized placebo-controlled trial [147]. However, its side effects on insulin resistance, increased pruritus severity increased and dyslipidemia, may hinder its clinical application [148,149,150]. A novel FXR agonist, TERN-101 developed by Eli Lilly, reduces liver steatosis, inflammation, ballooning, and fibrosis in a murine model of NASH. TERN-101 was granted by US Food and Drug Administration (FDA) fast track status to treat NASH in 2019 and is now in a phase 2a study in NASH patients.

Other potential approaches to reduce liver Chol or to decrease Chol-mediated liver injury for the treatment of NASH and HCC include thyroid hormone receptor agonists [151], activation of the glucagon-like peptide-1 receptor (GLP-1R) [152], and genetic deletion or silencing of miR-122 [153]. Overall, there are very limited and conflicting data on their effects on liver histology and HCC progression. There is also limited evidence regarding the safety and efficacy of other lipid-lowering agents in patients with NASH. Further studies are needed to clarify the potential of these approaches to be exploited therapeutically.

## 7. Conclusions

NASH and NASH-associated HCC has become a worldwide health problem and will continue to increase with the growing epidemic of obesity and insulin resistance in the future. NAFLD and HCC is strongly associated with obesity, insulin resistance and the metabolic syndrome and directly attributed to changes in lifestyle, especially dietary patterns and sedentary behavior. Western Diet and Chol contained in the diet exacerbate hepatic accumulation of free Chol, resulting in the toxic effects on the liver and altered Chol metabolism. Focusing on lifestyle modifications is necessary for HCC prevention. Developing better mouse models to reflect the human NASH and HCC signatures are needed to understand the molecular mechanisms of disease pathogenesis and explore the new treatment strategy.
